# Primary Pulmonary Mucinous Cystadenocarcinoma: A Case Report

**DOI:** 10.1155/2011/562026

**Published:** 2011-03-29

**Authors:** Andreas Efstathiou, Christos Asteriou, Nikolaos Barbetakis, Dimosthenis Miliaras, Athanassios Kleontas, Christos Karvelas, Miltiadis Lalountas

**Affiliations:** ^1^Cardiothoracic Surgery Department, Theagenio Cancer Hospital, Al. Symeonidi 2, 54007 Thessaloniki, Greece; ^2^Laboratory of Histology and Embryology, Medical School, Aristotle University of Thessaloniki, 54636 Thessaloniki, Greece; ^3^2nd Propedeutical Department of Surgery, Hippokration General Hospital, Aristotle University of Thessaloniki, Konstantinoupoleos 49, 54642 Thessaloniki, Greece

## Abstract

Primary pulmonary mucinous cystadenocarcinoma (PMCAC) is an extremely rare cystic neoplasm. A case of a 56-year-old male with a cystic lesion of the right lower lobe is described. Preoperative fine needle aspiration cytology and bronchoscopy were inconclusive. The patient underwent a formal right lower lobectomy and mediastinal lymph node dissection. Diagnosis was established intraoperatively. The biological behavior of primary PMCAC is unknown. Therefore, careful long-term follow-up is considered necessary because of lack of experience globally.

## 1. Introduction

Primary pulmonary mucinous cystadenocarcinoma (primary PMCAC) belongs to a group of cystic neoplasms characterized by extracellular mucin secretion. These mucinous neoplasms are histologically similar to well-recognized mucinous tumors of the ovary, breast, and pancreas [1]. Although it is considered malignant, its biological behavior is still under study. The incidence of this lesion seems to be unknown. Very few cases have been published in the English literature. Preoperative diagnosis is very difficult. Diagnosis is established in all cases by histopathology of the resected tumors, although fine needle aspiration (FNA), bronchial washings, brushings or biopsies may help preoperatively [2, 3]. Identification of this kind of lesion is very important because of presumed differences in management and prognosis.

## 2. Case Presentation

**Figure 1:**
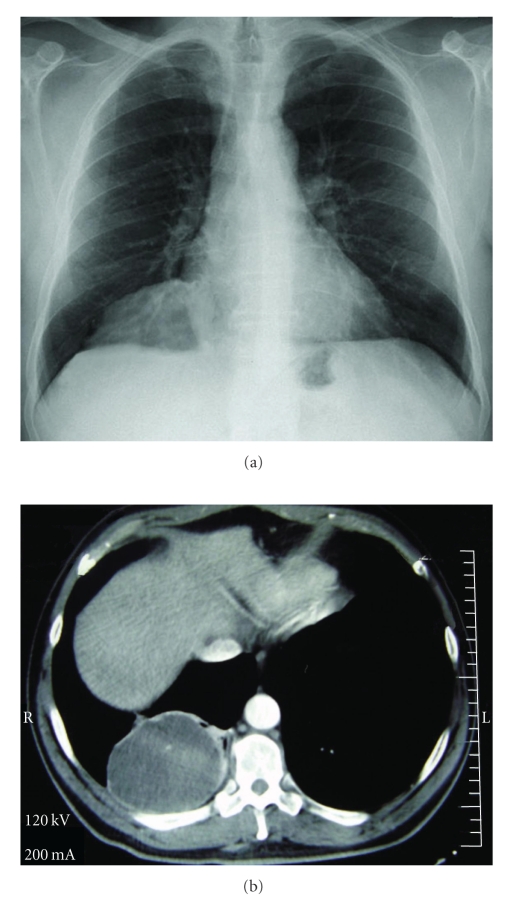
Primary PMCAC. (a) Chest X-ray revealing a solitary tumor in the right lower lobe of the lung. (b) Computed Tomography scan of the thorax. A large cystic well-demarcated mass in the right lower lobe of the lung is detected (19 Hounsfield Units).

A 56-year-old male presented with a complaint of chest pain. His medical record was clear, except that he was a heavy smoker (60 cigarettes/day for 38 years). Clinical examination revealed diminished breath sounds in the right hemithorax. A chest X-ray showed a large mass in the right lung (Figure 1(a)). Computed Tomography (CT) of the chest revealed a tumor originating in the right lower lobe, measuring 9.3 × 7.5 cm (Figure 1(b)). The lesion had a low attenuation (19 Hounsfield Units), and tiny, mildly enhancing septa was recognized. Mediastinal lymphadenopathy was not detected, while further brain and abdomen CT scans were negative. 

Bronchoscopy was negative for endobronchial growth, and CT-guided FNA cytology failed to establish a tissue diagnosis. The patient underwent a right posterolateral thoracotomy, and a right lower lobectomy was performed. Frozen sections of the specimen revealed a cystic mucinous mass with fibrous walls containing abundant mucinous material. Cells suspicious for malignancy were detected inside the mucin. The operation, therefore, was completed with a radical mediastinal lymph node dissection. The patient was discharged on the 11th postoperative day. No complications were observed. 

**Figure 2:**
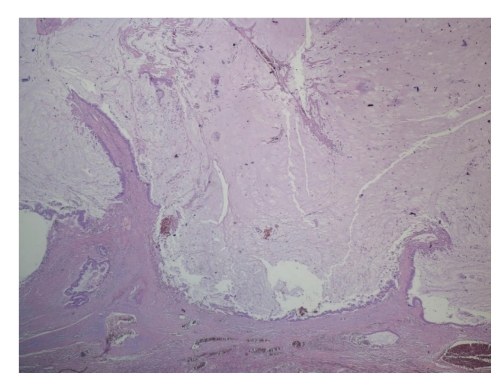
Primary PMCAC. The tumor consists of a large cystic space filled with mucin and lined by columnar neoplastic epithelium. (H&E, 25X).

The excised lobe contained a cystic tumor filled with mucin, 9.3 cm in greater diameter. Microscopic examination revealed a well-differentiated mucinous cystadenocarcinoma. The tumor consisted of a large cystic space filled with mucin, and many smaller cysts along its wall (Figure 2). The cystic spaces were lined by one or more layers of columnar, mucous-secreting cells with atypia, and few mitoses. The mucin pools were frequently dissecting through the surrounding stroma. In addition, small irregular groups of neoplastic cells were found to invade the surrounding stroma. The visceral pleura and the surgical margin of the lobar bronchus were free of tumor. All twenty-two removed lymph nodes (interlobar, hilar, and ipsilateral mediastinal) were also negative. In order to exclude the possibility of a metastatic adenocarcinoma, immunohistochemical investigation followed. The tumor cells were positive for cytokeratin 7 and negative for cytokeratin 20 and Thyroid Transcription Factor-1 (TTF-1). Retrospective review of both bronchoscopic and FNA findings suggests that a correct preoperative diagnosis was impossible.

The patient is currently been followed up closely. No signs of local or distal recurrence were observed during 6-months period.

## 3. Discussion

Primary PMCAC is a very rare entity. It was first described in 1978 by Sambrook Gowar [4]. It is considered to be the pulmonary counterpart of similar tumors in the ovary, breast, and pancreas. In the majority of the published cases, the patients were asymptomatic, and the tumor was discovered incidentally by performing a chest X-ray for other reason. Hemoptysis, recurrent bronchitis, and exacerbation of chronic obstructive pulmonary disease in three cases were reported as the initial clinical manifestation [5]. Thoracic pain was the chief complaint in our case.

Radiologically, some authors mention CT characteristics which can raise suspicion for the diagnosis. These include a uniform low attenuation cystic lesion with focal thickening and enhancement of the walls and septa [6]. CT images of our patient fit perfect in the above description. Furthermore, congenital diseases such as pulmonary sequestration, bronchogenic cyst, or bronchocele, infectious causes like hydatid cyst or abscess, and some types of other cystic neoplasms, primary or metastatic, complete the radiological differential diagnosis [7].

The normal bronchial mucus is 95–98% water and 2–5% solids, and the majority of the solid component is formed by glycoproteins. Due to its high water content, mucus has CT attenuation and Magnetic Resonance Imaging (MRI) signal similar to those of water and do not enhance after injection of intravenous contrast medium; however, a chronic entrapped mucus collection can undergo a gradual transformation. The increased production of mucus protein is accompanied by a gradual reabsorption of water with a decrease in free water content and an increase in protein concentration. Protein concentration influences the T1 relaxation time, and an elevation in protein concentration may cause impressive shortening of the T1 relaxation time. Mucus with a protein level >9,000 mg/dL has high signal intensity on T1-weighted images. The detection of mucus within a mediastinal or pulmonary lesion is important because it permits shortening of the list of differential diagnoses [8].

It has been well known that most malignant tumors have a higher rate of glucose metabolism than normal tissue. However, 18Fluoro-2-deoxy-D-glucose (FDG) uptake has a wide variation in tumor glucose metabolic rate, depending on the histologic type and aggressiveness of the tumor. Because FDG behaves as an analog of glucose, its distribution closely follows that of glucose-metabolizing cells and organs. Most malignant tumors have a higher rate of glucose metabolism than normal tissue, therefore, exhibit a higher FDG uptake than background tissue. Using FDG Positron Emission Tomography (PET) a positive correlation between tumor FDG uptake and cellularity can be proved, but a negative correlation with the amount of mucin is also expected. In case of PMCAC hypocellular lesion with abundant mucin, it is possible that little FDG uptake on integrated PET/CT be shown [9]. In addition, Yap et al. mentioned that noninvasive feature of tumor made negative FDG PET findings of tumor as well as mucin [10].

The histopathological differential diagnosis of primary PMCAC includes primary lesions of the lung and metastatic tumors from other organs, especially the ovary, breast, and pancreas [11]. Well-differentiated mucinous bronchoalveolar carcinoma is usually solid and has the ability to develop in association with a preexisting lung cavity. In mucoepidermoid carcinoma squamous and intermediate cells in combination are almost always present. Solitary metastatic mucinous tumors can mimic primary PMCAC; therefore, full investigation of the organs that may develop this kind of carcinoma is obligatory [12]. Mucinous cystadenocarcinoma of the lung represents an entity with two distinct clinicopathologic and immunophenotypic variants: the goblet cell-type, presenting a more indolent clinical behavior and frequently coexpressing markers of intestinal and pulmonary differentiation and the more aggressive signet-ring cell type, which retains only markers of pulmonary origin [13].

Nonneoplastic lesions that enter the differential diagnosis include developmental bronchogenic cysts and congenital adenomatoid malformations. Both can have foci of mucinous epithelium but more commonly are lined by ciliated columnar epithelium, in contrast with the purely mucinous epithelium of mucinous cystic tumors. Developmental bronchogenic cysts are extrapulmonary, located in the midline, and have a fibromuscular wall containing cartilage and seromucinous glands [5]. 

Preoperative diagnosis is very difficult to establish. Both FNA cytology and transbronchial lung biopsy seem inadequate, mainly because the malignant cells are floating inside the mucin. Only in two cases were the authors able to reach preoperative diagnosis by using FNA cytology [2, 3]. In our case both bronchoscopy and CT-guided FNA proved to be inefficient. As a result the surgical approach was mandatory. Frozen section revealed atypical cells inside the mucin. Their characteristics were compatible with a primary PMCAC.

The biological behavior of primary PMCAC is generally unknown. Although it is considered a malignant tumor, it seems not to have malignant potential of high degree. In the only case where recurrence occurred, margin negative wedge resection was performed at first. Due to the small number of reported cases in the literature, the tumor's developing pattern is not yet completely clarified. It seems, however, that local extension is more likely than distal metastases. Therefore, formal anatomic lobectomy for early stage tumors, in those patients who can physiologically withstand it, according to GN Mann et al, is probably the correct surgical approach [14]. The role of chemotherapy and radiotherapy is difficult to define because of the small number of cases and unclear natural history. Based on that, close long-term follow-up is obligatory.

##  Consent

Written informed consent was obtained from the patient for publication of this paper and accompanying images. A copy of the written consent is available for review by the Editor-in-Chief of this journal.

##  Conflict of Interests

The authors declare no conflict of interests.
